# Torsade de Pointes: are psychotropic drugs at the heart of the matter? A retrospective case-control study led at the Montreal Heart Institute

**DOI:** 10.1192/j.eurpsy.2023.1129

**Published:** 2023-07-19

**Authors:** E. Laszlo, S. Cyr, R. Tadros, M.-C. Guertin, C. Fiset, J. Brouillette

**Affiliations:** 1 Centre de recherche de l’Institut de Cardiologie de Montréal, Montreal Heart Institute; 2 Faculty of Pharmacy, Université de Montréal; 3 Montreal Health Innovations Coordinating Center, Montreal Heart Institute; 4 Department of psychiatry and addiction, Université de Montréal, Montréal, Canada

## Abstract

**Introduction:**

Psychotropic drugs are the first-line medications in the treatment of psychosis, bipolar, anxiety and depressive disorders. Some of these psychoactive agents are suspected to be linked to rare, but lethal, ventricular arrhythmias, known as Torsade de Pointes (TdP). Most of the studies found an association between these classes of psychiatric agents and a prolongation of the corrected QT interval. However, QTc prolongation remains an imperfect, though well-established marker of risk for TdP and little is known about the relation between psychotropic drugs and TdP. Some physicians hence refrain from prescribing psychotropic medications to their patients for fear of cardiac adverse events, which can severely undermine the management of underlying psychiatric conditions. It is thus crucial to evaluate the relation between psychotropic medication use and the occurrence of TdP.

**Objectives:**

The primary objective of this study is to assess the relative contribution of psychotropic medications (antidepressants, antipsychotics) among all TdP risk factors (e.g. sex, hypokalemia, antiarrhythmic drug use). We hypothesize that psychotropic drug use will indeed be associated to TdP, but that this association is negligeable compared to other TdP risk factors.

**Methods:**

A retrospective case-control study (1 :3 ratio) of patients hospitalized at the Montreal Heart Institute was carried out (n=444).

**Results:**

Antidepressant and antipsychotic medication use proportions among the cases are 27% and 12% respectively, compared to 17% and 5% in controls (p= .018407 and p= .016326). In our study, patients who take antidepressants [OR=1.83; 95% CI 1.10-3.04] or antipsychotics [OR=2.47; 95% CI 1.16-5.26] are more likely to experience TdP. Patients with a psychotropic polypharmacy are also more prone to TdP [OR=5.67; 95% CI 2.58-12.42]. However, cases are also significantly more likely (p=.000281) to take concomitant medications associated with QTc prolongation (based on CredibleMeds, July 2022 list). Female sex [OR=2.40; 95% CI 1.55-3.71], hypokalemia [OR=3.46; 95% CI 1.65-7.26], kidney failure [OR=1.61; 95% CI 1.05-2.48], a QTc interval greater or equal to 500 ms [OR=5.89; 95% CI 3.59-9.65] are also associated with TdP.

**Conclusions:**

In this study, psychotropic drug use is indeed associated to TdP. Further analyses, *i.e.* multivariate logistic regressions, will determine psychotropic drugs’ relative contribution among the identified risk factors for TdP.

**Disclosure of Interest:**

None Declared

